# Chondroitin sulfate proteoglycans regulate the growth, differentiation and migration of multipotent neural precursor cells through the integrin signaling pathway

**DOI:** 10.1186/1471-2202-10-128

**Published:** 2009-10-21

**Authors:** Wen-Li Gu, Sai-Li Fu, Yan-Xia Wang, Ying Li, He-Zuo Lü, Xiao-Ming Xu, Pei-Hua Lu

**Affiliations:** 1 Department of Neurobiology, Shanghai Jiao Tong University School of Medicine, Shanghai 200025, PR China; 2 Department of Clinical Laboratory, No.9th People's Hospital, Shanghai Jiao Tong University School of Medicine, Shanghai 200011, PR China; 3 Spinal Cord and Brain Injury Research Group, Stark Neurosciences Research Institute, Department of Neurological Surgery, Indiana University School of Medicine, Indianapolis, IN 46202, USA

## Abstract

**Background:**

Neural precursor cells (NPCs) are defined by their ability to proliferate, self-renew, and retain the potential to differentiate into neurons and glia. Deciphering the factors that regulate their behaviors will greatly aid in their use as potential therapeutic agents or targets. Chondroitin sulfate proteoglycans (CSPGs) are prominent components of the extracellular matrix (ECM) in the central nervous system (CNS) and are assumed to play important roles in controlling neuronal differentiation and development.

**Results:**

In the present study, we demonstrated that CSPGs were constitutively expressed on the NPCs isolated from the E16 rat embryonic brain. When chondroitinase ABC was used to abolish the function of endogenous CSPGs on NPCs, it induced a series of biological responses including the proliferation, differentiation and migration of NPCs, indicating that CSPGs may play a critical role in NPC development and differentiation. Finally, we provided evidence suggesting that integrin signaling pathway may be involved in the effects of CSPGs on NPCs.

**Conclusion:**

The present study investigating the influence and mechanisms of CSPGs on the differentiation and migration of NPCs should help us to understand the basic biology of NPCs during CNS development and provide new insights into developing new strategies for the treatment of the neurological disorders in the CNS.

## Background

Neural precursor cells (NPCs) are defined by their ability to proliferate, self-renew, and retain the potential to differentiate into neurons and glia. In recent years, NPCs have garnered much attention because they can reveal clues about the nervous system development [1] and possibly be used as promising therapeutic agents. NPC transplantation has the potential to ameliorate various neurological diseases and injuries, such as Parkinson's disease, Huntington's disease, stroke, and traumatic brain injury, and lead to partial functional recovery [2,3]. It has been reported that the endogenous NPCs in the brain reside in a specialized compartment termed "niche" which is a specialized microenvironment composed of soluble factors, membrane-bound molecules and extracellular matrix (ECM) constituents that may regulate NPC function [4,5].

The ECM environment in the central nervous system (CNS) is responsible for a large number of regulatory functions both during development and adulthood. It provides signals for cell growth, differentiation and migration. These activities are critical for the development of CNS and disruptions of ECM interactions can cause severe developmental defects [6]. Several groups have shown that ECM affects rodent NPCs proliferation, migration, and differentiation both in vitro [7,8] and vivo [9], and signals from the well-defined surrounding matrix are involved in the regulation of NPCs. Recently, there is growing evidence that the functions of ECM on NPCs are mediated through the activation of integrin and MAP kinase pathways [10-12]. Integrins are a major group of cell-surface receptors for both ECM and cell-surface molecules. They are the primary mediator of neural cell behavior on ECM components and control various nervous system cell functions including survival [13], migration [14,15], neurite outgrowth [16], and myelination [17]. However, the mechanism by which integrin regulates NPC behavior remains largely unexplored.

Chondroitin sulfate proteoglycans (CSPGs) are prominent components of the ECM in the CNS and are assumed to play a particularly important role in controlling neuronal differentiation and development. During development, they appear to function in axon guidance by restricting axon growth to inappropriate targets [18], as well inhibit the migration of neural crest cells [19]. Later in development and in adulthood, they have been shown to regulate the neuronal plasticity by forming perineuronal nets around synapses [20,21]. In damaged nervous system, they exert growth-inhibitory activities in the glial scar such as hampering axon regeneration or compensatory sprouting [22-24]. Though it was reported that CSPGs were expressed on NPCs in culture and could be secreted into media [25], little is known about their physiological role in the development of NPCs in the CNS.

It has been well known that the application of Chondroitinase ABC (Chase ABC) can promote axonal regeneration by digesting CSPGs after spinal cord injury [26-28], and NPC transplantation is another promising strategy for spinal cord injury therapy. Recently, researchers found that the combination of the two strategies could significantly induce the outgrowth of GAP-43^+ ^fibers at the lesion epicenter of injured spinal cord, compared with either therapy alone [25]. However, an important detail has not been addressed, i.e. were the proliferation and differentiation of transplanted NPCs affected by their exposure to Chase ABC?

In the present study, we used Chase ABC to eliminate the effects of endogenous CSPGs and determined whether such elimination resulted in abnormal growth properties of NPCs. Furthermore, we also examined whether integrin signal pathways were involved in mediating the biological effects of CSPGs on NPCs.

## Methods

### Isolation and cultivation of NPCs

Neural precursor cells were prepared according to the method of Hu [29], with appreciable modifications. Briefly, NPCs were isolated from the embryonic brain of the E16 Sprague-Dawley rat. After removal of the amnion and dura, the brain was dissected out in a sterile dish containing ice-cold Leibovitz's L15 medium (Invitrogen, Grand Island, NY, USA), and dissociated by gentle mechanical pipeting through fire-polished Pasteur pipettes to achieve single-cell suspensions. The dissociated cells were filtered through a nylon mesh of 70 μm (Falcon, NJ, USA), seeded into a T25 Corning tissue culture flask (Corning Inc., Corning, NY, USA) containing growth medium at a density of 1 × 10^5^cells/ml, and incubated in a humid atmosphere containing 5% CO_2 _at 37°C. The culture medium for NPCs contained: 1 × Dulbecco's Modified Eagle's Medium/F12 (DMEM/F12, Invitrogen), 1 × N2 (Invitrogen), 2% B27 (Invitrogen), 0.06% glucose, and 2 mM glutamine (Invitrogen), 1.34 mM bicarbonate sodium, 0.5 mM HEPES, 2 μg/ml heparin (all from Sigma, St.Louis, MO, USA), and freshly added 20 ng/ml epidermal growth factor (EGF, Sigma) and 20 ng/ml bFGF (Invitrogen). After 3 or 4 days, the cells grew and developed into visible neurospheres of 50-200 cells/sphere; then they were collected and mechanically pipetted into single cells for passage. The NPCs of passage 2 were collected for the following assay.

To identify the expression of CSPGs on the NPCs, 50 μl dissociated cells in single-cell suspension were seeded onto laminin (10 μg/ml, Gibco) coated coverslips in 35-mm dishes at a density of 3 × 10^4 ^cells/coverslip, and cultured in NPC growth medium supplemented with EGF and bFGF. 48 hours after culture, the cells were fixed for immunostaining.

All embryonic rats were obtained from female pregnant SD rats bred in the Animal Care Facility at Shanghai Jiaotong University School of Medicine. All animal care was performed in accordance with the National Institutes of Health Guide for the Care and Use of Laboratory Animals (NIH Publications No. 80-23; revised 1996).

### Differentiation of NPCs

To induce the differentiation of NPCs, 65 μl dissociated cells in single-cell suspension were seeded onto poly-L-lysine (200 μg/ml, Sigma) coated coverslips in 35-mm dishes at a density of 5 × 10^4 ^cells/coverslip. Then, growth factors were removed from the growth medium, and 1% fetal bovine serum (FBS, Invitrogen) was added. The cultures were allowed to differentiate for 5 days in vitro before being fixed for immunostaining.

### [3H] Thymidine Incorporation Assay

NPCs of passage 2 were dissociated and transferred to 96-well plates (1.0 × 10^4^cells/100 μl/well), after that, another 100 μl of different concentrations (0.05-50 mU/mL) of Chondroitinase ABC (Chase ABC, Seikagaku, Tokyo, Japan) or 100 μl medium alone (control) were added to the wells, then cultured in medium with EGF and bFGF for 24 hours at 37°C containing 5% CO_2_. And 1 μCi/well [^3^H] thymidine (Amersham Piscataway, NJ, USA) was added to the cultures for the last 16 hours. The cells were harvested on fiberglass filters (Whatman, Kent, UK) using a cell harvester (Tomtec, CT, USA). Incorporated radioactivity was measured using standard scintillation techniques.

### Immunocytochemistry (ICH)

For floating neurospheres, they were harvested and fixed using 4% paraformaldehyde (PFA) in 0.01 M PBS (pH 7.4) at 4°C overnight and cryoprotected in PBS containing 30% sucrose, then embedded in OCT (Sakura FineTec Inc., Torrance, CA, USA) and sectioned with a cryostat. For cell cultures on the coverslips, they needed to be fixed with 4% PFA for 10 min at room temperature (RT) in advance, then washed and stored in 0.01 M PBS (PH7.4). Thereafter, the sections of neurospheres or cell cultures were blocked in 10% goat serum in PBS (for cell surface staining) or 0.3% Triton X-100-containing 10% goat serum in PBS (for intracellular staining) for 1 hour at RT, then sequentially incubated with the following primary antibodies overnight at 4°C: the monoclonal mouse antibodies IgM against CS-56 (1:200, Sigma) for CSPGs, the monoclonal mouse antibodies IgG against nestin (1:800, BD Pharmingen, San Jose, CA, USA) for NPCs, βIII-tubulin (1:800, Sigma) for neurons, glial fibrillary acidic protein (GFAP, 1:200, Sigma) for astrocytes, or the monoclonal mouse antibodies IgM against O4 (1:600, R & D, Minneapolis, MN, USA) and the monoclonal mouse antibodies IgG against myelin basic protein (MBP, 1:40, Siemens Inc, Oncogene Group, Cambridge, MA, USA) for oligodendrocytes. After being washed with PBS, sections and cell cultures were incubated for 60 min at 37°C with the appropriate secondary antibody: fluorescein isothiocyanate (FITC)-conjugated goat anti-mouse IgM (1:100, Santa Cruz Biotechnology, Santa Cruz, CA, USA), FITC-conjugated goat anti-mouse IgG (1:100, Sigma), rhodamine-conjugated goat anti-mouse IgM (1:200, Santa Cruz Biotechnology), and rhodamine-conjugated goat anti-mouse IgG (1:100, Sigma). After staining, the slides or coverslips were rinsed and mounted with Gel/Mount aqueous mounting media (Biomeda Corp., Foster City, CA, USA) containing Hoechst 33342 (1 μg/ml, Sigma), a fluorescent nuclear dye. The images were acquired using an Olympus BX60 microscope equipped with a digital camera and SPOT 4.0.1(G) software. For cell counts, at least five randomly selected fields with a total of more than 1000 cells were counted. For neurite outgrowth assay, the neurite lengths of a total of 50 neurons in each group from four independent experiments were measured with a Neurolucida system (MicroBrightField Inc., Colchester VT, USA). In all experiments, primary antibody omission controls were used to confirm the specificity of Immunocytochemistry.

### Western blot analysis

Dissociated NPCs were seeded into 60-mm dishes (Corning Inc.) and cultured in D/F12 medium containing 1% FBS for 5 days, with or without Chase ABC administration. Then cells were washed and lysed with a lysis buffer (50 mM Tris-HCl, 150 mM NaCl buffer, 1% NP-40, 0.5% sodium deoxycholate, 0.1% SDS, 1 mM EDTA, 1 mM sodium orthovanadate, 10 mM sodium fluoride, 4 μg/ml leupeptin, 1 μg/ml aprotinin and 100 μg/ml PMSF; all from Sigma). The supernatant was clarified by centrifugation at 16,000 g for 10 min at 4°C. The protein concentration of the lysate was determined using a BCA Protein Assay kit (Pierce, Rockford, IL, USA). For western blotting, Protein samples containing an equal amount of protein (15 μg) were electrophoresed on SDS-polyacrylamide gels, and transferred to polyvinylidene difluoride filters (Millipore, Bedford, MA, USA). The filters were blocked with 5% non-fat dry milk in Tris-buffered saline (TBS) for 1 hour at RT and then incubated overnight at 4°C with primary antibodies (in TBST-5% BSA) including GFAP (1:1000, Sigma), βIII-tubulin (1:2000, Sigma), MBP (1:100, Oncogene) as markers for the differentiated neural cells, and GAPDH (1:8000, Kangchen Bio-Tech) as an internal control. After being rinsed with TBST, the membranes were incubated with the secondary antibody HRP-conjugated goat anti-mouse IgG (1:1000, R & D) for 1 hour at RT. To visualize the immunoreactive proteins, the ECL kit (Pierce) was used, following the manufacture's instructions.

### FACS analysis

For flow cytometric analysis of DNA content, NPCs were cultured in medium alone or incubated with Chase ABC at 37°C for 48 hours, and approximately 10^6 ^cells were harvested, washed with ice-cold PBS, then fixed with 70% ethanol at 4°C overnight. After that, fixed cells were washed in PBS and re-suspended in a staining solution containing 50 μg/mL propidium iodide (BD Pharmingen) in 0.1% sodium citrate, 0.1% NP-40, and 12.5 μL ribonuclease (RNase) 1 mg/mL. Cells were incubated for 15 min at 37°C, then kept in the dark and analyzed using a FACSCalibur (BD Biosciences, Mountain View, CA, USA) with excitation at 480 nm and the fluorescent signal was collected at 585 nm. Cell cycle populations in the G1-, S-, and G2-phases were quantified with the ModFit LT v3.0 software.

### Cell migration assay

Neurospheres of passage 2, grown for 3 days in growth medium supplemented with EGF and bFGF, were uniformly seeded on the well bottoms of a sterile 35-mm dish, which were in advance coated with Laminin (10 μg/mL, Invitrogen). Neurospheres were plated at low density to ensure large distance between individual spheres and incubated with Chase ABC at 37°C in NPCs growth medium containing EGF and bFGF. The control groups were grown under identical conditions except for Chase ABC. At three selected time point, i.e. 2, 4.5 and 8 hours, the cultures were observed and photographed by an inverted microscope (IX70, Olympus, Tokyo, Japan) equipped with a CCD camera (DP70, Olympus). Three samples were evaluated for each set of conditions, and a total of more than 30 neurospheres at each selected time point were randomly captured and imaged. The average migration distance of the neurospheres was expressed by the diameter of the spheres surrounded by the rim of migrating cells, based on previous reports [7,15], and represented as the mean of the straight-line measurement from the center of the neurosphere to the farthest defined edge of the migratory cell boundary in four vertical directions using SPOT 4.0.1(G) software.

### Integrin blocking test

For neurosphere growth test, NPCs were divided into 4 groups: control group in regular growth medium, Chase ABC treatment group, integrin blocking group which were incubated with Echistatin (Ech, Sigma), the most potent known inhibitor of integrin function, and combined group with Chase ABC and Ech. The NPCs cultured on the uncoated dishes in the growth medium containing EGF and bFGF, were observed and photographed 24 hours after culture, by an inverted microscope (IX70, Olympus) equipped with a CCD camera (DP70, Olympus). For differentiation experiments, there were 3 groups: control group, Chase ABC group, and Chase ABC+Ech group. The dissociated NPCs of each group were cultured on the poly-L-lysine coated coverslips in differentiation medium with 1% FCS for 5 days, then, fixed with 4% PFA for MBP immunostaining as described above.

### Statistical analysis

Data were presented as mean ± standard error of the mean (SEM) values. Statistical analysis was performed by SPSS 10.0 and One-way analysis of variance (ANOVA) with post hoc Tukey LSD test or two-tailed independent sample T-Test was used to determine statistical significance. A P-value less than 0.05 was considered statistically significant.

## Results

### Expression of CSPGs on NPCs in vitro

**Figure 1:**
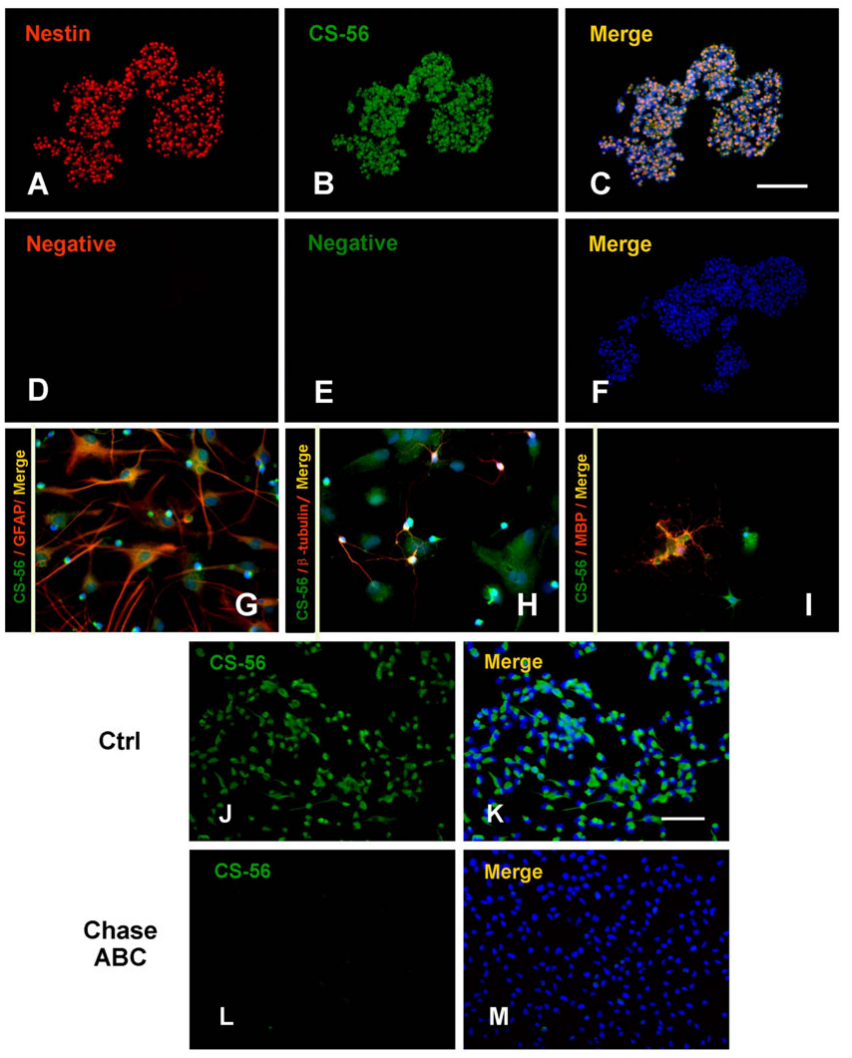
**CSPG Expression on NPCs in vitro**. (A-C) Immunofluorescence double staining of Nestin (A, red) and CSPGs (B, green) on neurospheres showed that almost all NPCs expressed CSPG (C, merge). The NPCs from E16 rat embryonic forebrains were cultured for 3 days in the NPC growth medium supplemented with EGF and bFGF. (D-F) The negative controls consisted of omission of the primary antibody. (G-I) The differentiated NPCs were double immunostained using anti-CS56 antibody (green) and anti-GFAP, βIII-tubulin and MBP antibodies (red) respectively. The result showed that CSPGs were expressed on GFAP^+ ^astrocytes (G), βIII-tubulin^+ ^neurons (H) and MBP^+ ^oligodendrocytes (I). (J-M) The expression of CSPGs was observed on the dissociated NPCs culture in the growth medium containing EGF and bFGF (J, K), but diminished after Chase ABC (50 mU/mL) treatment for 48 hours (L, M). Hoechst 33342 (blue) was used to stain the nuclei. Scale bars: A~F, 100 μm; G~M, 50 μm.

When dissociated NPCs from E16 rat embryonic forebrains were cultured in serum-free N2/B27 growth medium supplemented with EGF and bFGF, they proliferated rapidly to form floating neurospheres. The immunostaining of sectioned neurospheres indicated that nearly all cells within spheres expressed the intermediate filament protein (Nestin), a marker for NPCs (Fig. 1A). After removal of EGF and bFGF and addition of 1% FBS, the NPCs plated on the poly-L-lysine coated coverslips began to differentiate along three neural lineages into a mixture of βIII-tubulin^+ ^neurons, GFAP^+ ^astrocytes and O4^+ ^or MBP^+ ^oligodendrocytes.

Subsequently, the expression patterns of CSPGs on NPCs and their lineage cells, including neurons, astrocytes, and oligodendrocytes were tested by double immunofluorescence staining with anti-CS56 antibody (special to Chondroitin sulfate moiety of CSPGs) and anti-Nestin, GFAP, βIII-tubulin and MBP antibodies respectively. The results showed that CSPGs were constitutively expressed on Nestin^+ ^NPCs (Fig. 1A-C) and also expressed on differentiated GFAP^+ ^astrocytes (Fig. 1G), βIII-tubulin^+ ^neurons (Fig. 1H) and MBP^+ ^oligodendrocytes (Fig. 1I). Moreover, the expression of CSPGs could also be observed on the dissociated NPCs cultured on the coverslips in the growth medium containing EGF and bFGF (Fig. 1J, K), but diminished after Chase ABC (50 mU/mL) treatment for 48 hours (Fig. 1L, M). As anti-CS56 antibody directed against the Chondroitin sulfate glycosaminoglycan (CS-GAG) chains of CSPGs, this result indicated that Chase ABC treatment could result in complete removal of the CS-GAG chains from the surface of NPCs.

### Effects of Chase ABC on the morphology of NPCs expanded in spheres

To explore the possible roles of endogenous CSPGs in the growth and differentiation of NPCs, we examined the effects of CSPGs on the morphology, proliferation, migration and differentiation of cultured NPCs by treating them with Chase ABC which abolished the function of endogenous CSPGs by the removal of CS-GAG side chains bounded on their core proteins.

**Figure 2:**
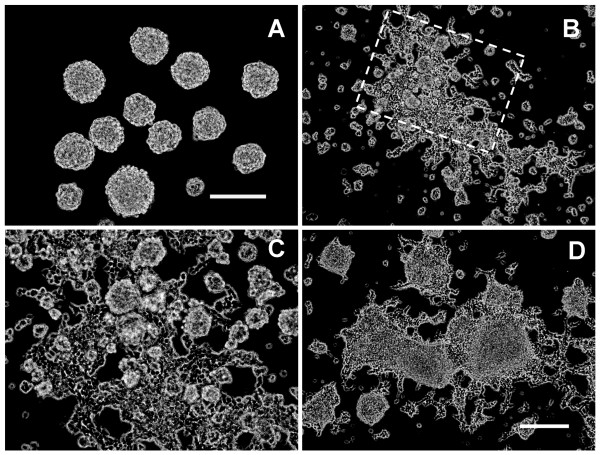
**Chase ABC induced morphological changes of NPC spheres**. (A) A phase-contrast photomicrograph shows floating neurospheres derived from disassociated NPCs cultured for 24 hours in growth medium supplemented with EGF and bFGF;. (B-D) Exposure of neurospheres to Chase ABC for 24 hours (B, C) and 48 hours (D) induced the neurospheres to attach to the bottom of the wells and migration of cells outward to form irregular cell clusters. Boxed area in (B) is viewed in (C) at higher magnification, which showed that many NPCs migrated out of the spheres and emerged irregular small processes from the cell body. Scale bar: A and C, 100 μm; B and D, 200 μm.

First, we examined the effect of Chase ABC on the morphology of NPCs expanded in spheres. When dissociated NPCs were cultured in serum-free N2/B27 growth medium supplemented with EGF and bFGF, they proliferated rapidly to form floating neurospheres 24 hours later (Fig. 2A). However, the NPCs in the completely identical growth medium, tended to turn flat and attach to the untreated bottom of the culture wells after Chase ABC (10 mU/mL) treatment for 24 hours, and only a few of them could still form small floating neurospheres, though the NPCs under treatment of Chase ABC could still proliferate rapidly during the initial culture. (Fig. 2B). Meanwhile, some NPCs migrated out of the spheres, the globular cells began to elongate, and irregular and small processes emerged from the cell bodies (Fig. 2C). As time went on, this tendency was more obvious. Most of neurospheres were adherent to the bottom of the well and spread outward to form irregular cell clusters which were surrounded by a layer of reticular, spindle-like cells in the periphery (Fig. 2D). In addition, the effects on the morphological change of neurospheres could occur in a wide concentration range of Chase ABC, from 0.5 to 50 mU/mL, even at a concentration of 0.1 mU/mL, although after administration of 50 mU/mL of Chase ABC, the NPCs attached to the bottom of well more quickly than that treated with lower doses of Chase ABC. It indicates that CSPGs, either expressed on the surface of NPCs or secreted into the ECM, may play an important role in maintaining the shape of NPCs and further, the fate of neurospheres.

### Effects of Chase ABC on the proliferation of NPCs

**Figure 3:**
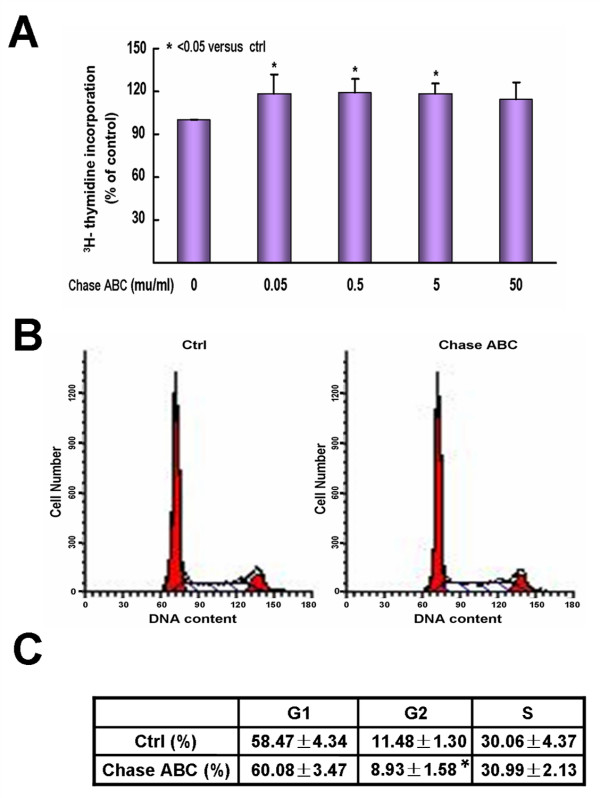
**Effects of Chase ABC treatment on the proliferation of NPCs in vitro**. The dissociated NPCs were cultured in growth medium containing EGF and bFGF for 24 or 48 hours with or without Chase ABC. (A) Bar graph shows that Chase ABC treatment for 24 hours enhanced NPC proliferation at a dose of 0.05 to 5 mU/mL, measured as percentage of control radioactive counts (cpm value). Data represent mean ± SEM of three independent experiments performed in triplicate. * P < 0.05. (B-C) Cell cycle was analysed by FACS, 48 hours after Chase ABC treatment on NPCs. (B) Histograms indicate the DNA distribution in control and Chase ABC treated groups. (C) The percentages of cell populations in G1-, G2-, and S-phases were derived from DNA histograms (B) by a deconvolution analysis. The results showed that the treatment of Chase ABC barely affected the cell cycle except for the G2-phase. Data shown were mean ± SEM of four independent experiments. * P < 0.05.

Second, we studied whether Chase ABC treatment would influence the proliferation of NPCs by [^3^H] thymidine incorporation. The disassociated NPCs were cultured in the NPC growth medium containing EGF and bFGF and examined after 24 hours exposure to different concentration of Chase ABC or without. The results showed that administration of Chase ABC at a dose of 0.05 to 5 mU/mL resulted in a significant increase in [^3^H] thymidine incorporation, about 19-20% higher than the control (P < 0.05). Treatment of higher dose of Chase ABC, i.e. 50 mU/mL, did not bring more striking effects and showed no significant difference compared to the control (P > 0.05) (Fig. 3A).

To further elucidate the influence of Chase ABC on the proliferation of NPCs, we subsequently examined changes in cell cycle after Chase ABC treatment by flow cytometric analysis of the DNA content (propidium iodide staining). We used 5 mU/mL of Chase ABC as treatment group which had shown higher proliferation in the [^3^H] thymidine incorporation test. However, after treatment for 48 hours, significant changes did not occur in cell populations at the G1- and S-phases whereas the cells in G2-phase decreased significantly (8.93 ± 1.58%) compared to the control (11.48 ± 1.30%, P < 0.05) (Fig. 3B-C). We also observed changes in cell cycle 24 hours after the Chase ABC treatment, which were similar to that observed in the cell population analysis (data not shown). Therefore, interfering with the function of endogenous CSPGs with Chase ABC did not affect the cell cycle of NPCs except the cells in G2-phase.

### Chase ABC treatment enhanced the migration of NPCs out of spheres

**Figure 4:**
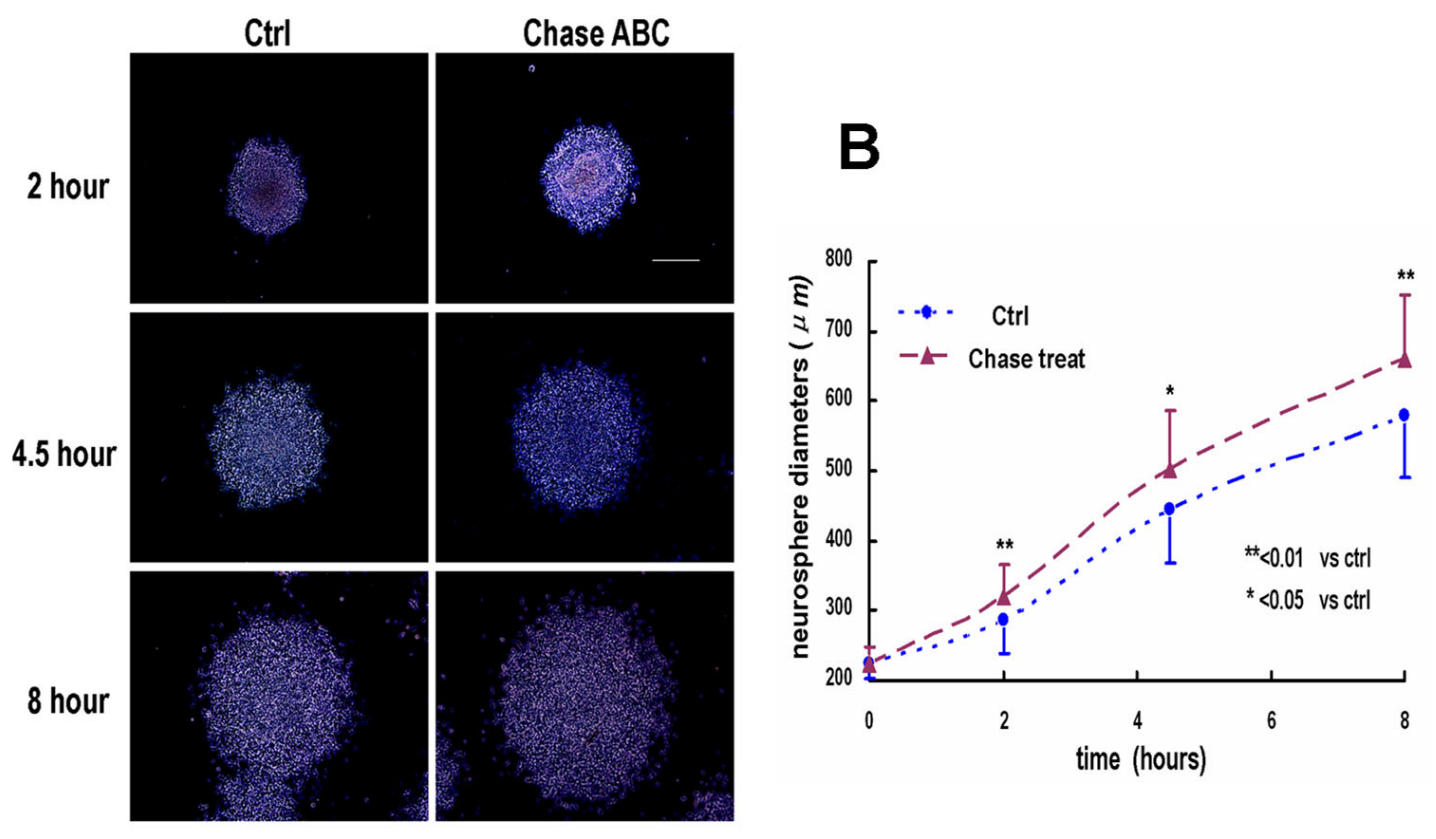
**Migration of NPCs out of neurospheres was enhanced by treatment of Chase ABC**. (A) Neurospheres were imaged at 2, 4.5 and 8 hours after plating into the culture wells with or without Chase ABC. (B) The quantification of NPC migration at 2, 4.5 and 8 hours were performed by measuring the diameters of the spheres surrounded by rim of migrating cells, and the results were showed in a time course graph. Data represent the mean ± SEM obtained from three independent experiments with a total of more than 30 neurospheres examined in each time point. * P < 0.05, ** P < 0.01. Scale bars: 200 μm.

To investigate whether Chase ABC treatment would affect the migration of NPCs, we then assessed NPCs motility by using a previously described bulk neurosphere assay [7,15]. The neurospheres, cultured in growth medium for 3 days, were uniformly seeded into wells coated with Laminin and treated with or without Chase ABC (50 mU/mL). The neurospheres attached to the well bottom within 1 hour and individual NPCs migrated rapidly away from the spheres, resulting in a rim of cells that formed a monolayer around the spheres, especially apparent on the periphery of the attached neurospheres. We selected 3 time points, i.e. 2, 4.5 and 8-hour to examine the spheres' motility (Fig. 4A), because after that, most of the cells from the spheres migrated outward too far to be completely recorded in a field of view of a microscope.

The average migration distance of the NPCs in spheres was expressed by the diameter of the spheres including the rim of migrating cells. The average diameters at the three selected time points were 320.0 ± 45.1 μm, 501.9 ± 83.4 μm and 660.0 ± 93.0 μm respectively in Chase ABC treated wells compared to 287.4 ± 47.8 μm, 444.9 ± 76.4 μm and 579.6 ± 89.7 μm, respectively, in controls. Chase ABC treatment promoted greater migration of NPCs than that of the control at all selected time points (P < 0.01 at 2-hour and 8-hour; P < 0.05 at 4.5-hour) (Fig. 4B). This indicates that interfering with the function of CSPGs on NPCs enhances the migration of NPCs out of spheres. It also indicates that CSPGs is inhibitory to the migration of NPCs and may contribute to the formation of NPC spheres.

### Effects of Chase ABC on the differentiation of NPCs in vitro

**Figure 5:**
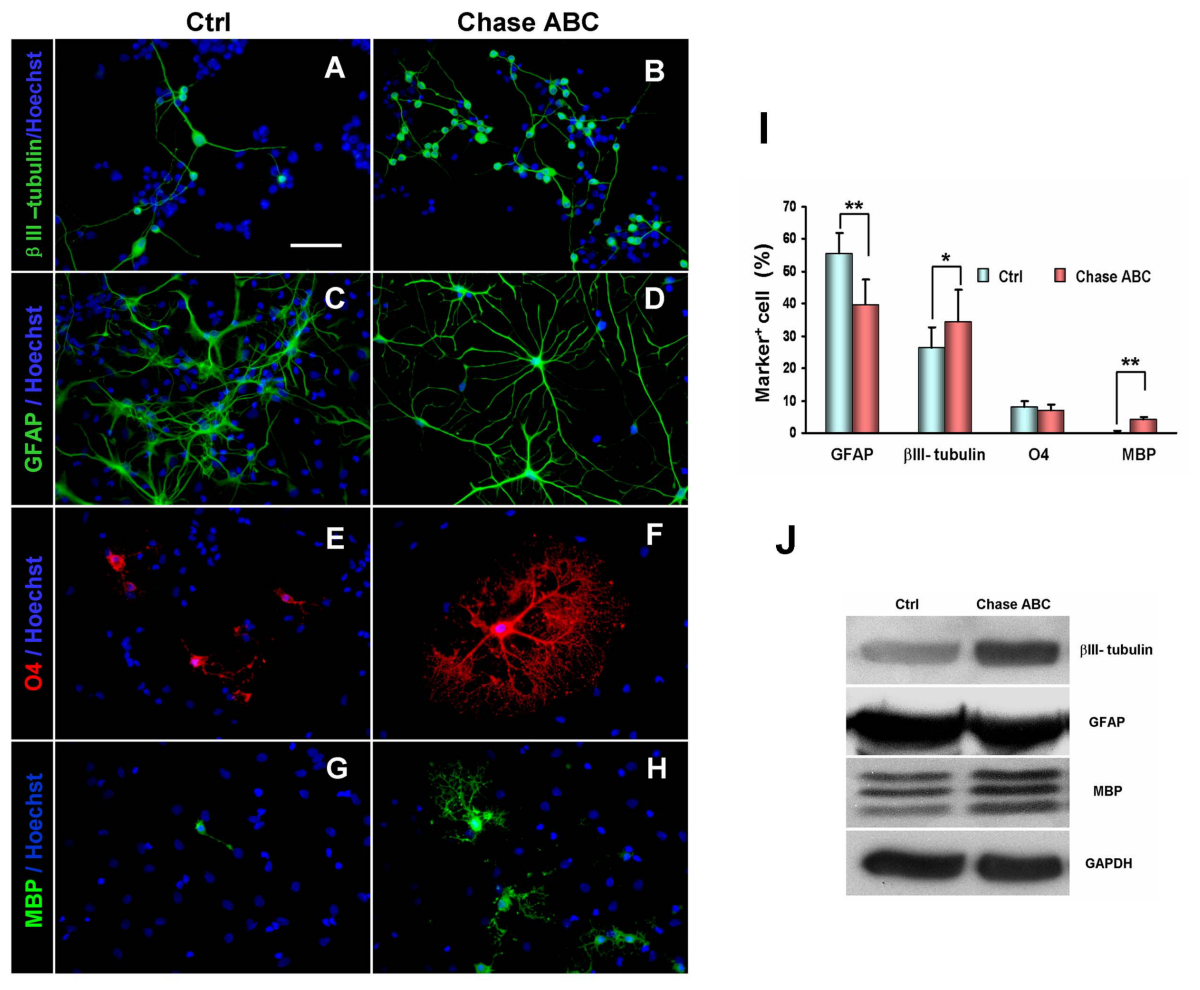
**Effects of Chase ABC on the differentiation of NPCs in vitro**. (A-D) Treatment of Chase ABC on NPCs for 5 days resulted in a substantial increase in the number of β-III tubulin^+ ^neurons (green, B) and decrease in the number of GFAP^+ ^astrocytes (green, D) as compared to the control (A and C, respectively). (E, F) No significant difference was found in the number of O4^+ ^(red) between the two groups. However, in the Chase ABC treated group, the O4^+ ^oligodendrocytes showed more mature morphology (F). (G, H) Chase ABC treatment brought about more mature MBP^+ ^oligodendrocytes (green, H) as compared to the control in differentiated NPCs. Hoechst 33342 was used as a nuclear marker for the estimation of cell numbers (A-H). Scale bar: 50 μm. (I) Quantitative data demonstrate that treatment of NPCs with Chase ABC resulted in a significant increase in the number of β-III tubulin^+ ^neurons (P < 0.05), decrease of GFAP^+ ^astrocytes (P < 0.01), no change of O4^+ ^oligodendrocytes (P *> *0.05) and increase of MBP^+ ^oligodendrocytes (P < 0.01). Data were shown as mean ± SEM of three independent experiments with a total of more than 1000 cells examined in each group. * P < 0.05, ** P < 0.01. (J) Western blot analysis shows that Chase ABC treatment resulted in a marked increase in the expression of β-III tubulin and MBP in differentiated NPCs, which were consistent with the immunostaining results. However, the expression of GFAP in the Chase ABC-treated group showed no obvious change.

To investigate whether Chase ABC treatment would affect the fate of NPCs, We next examined the differentiation of NPCs plated on poly-L-lysine-coated coverslips in differentiation medium. The results showed that the treatment of Chase ABC (50 mU/ml) on NPCs for 5 days in the presence of 1% FBS resulted in a significant increase of β-III tubulin^+ ^neurons (34.5% ± 9.9% vs 26.2% ± 6.6%, P < 0.05, Fig. 5A-B, I), and a decrease of GFAP^+ ^astrocytes (39.8% ± 7.9% vs 55.4% ± 6.4%; P < 0.01, Fig. 5C-D, I) compared to the control. Further more, we found that the morphology of GFAP^+ ^astrocytes generated from NPCs under Chase ABC treatment was a little different from those in the control, most of which exhibited type-II astrocytes morphology with stellate cell body, but owning longer and more highly branched processes than those seen in the control (Fig. 5D). Meanwhile, we also found that O4^+ ^oligodendrocytes exhibited more mature morphology in Chase ABC treated group than the control, although the percentage of O4^+ ^oligodendrocytes showed no statistical difference between the treated and control groups (7.03% ± 1.90% vs 8.02% ± 1.76%, P > 0.05, Fig. 5E, F, I). The typically mature morphology of oligodendrocyte from treated group was shown in Fig. 5F. When a more mature marker MBP was used, Chase ABC treatment significantly increased the percentage of highly branched MBP^+ ^mature oligodendrocytes (4.28% ± 0.66% vs 1.08% ± 0.63%, P < 0.01, Fig. 5G-I).

To further confirm the immunostaining results, we examined the expression of differentiation marker proteins (β-III tubulin, GFAP and MBP) with Western blot analysis. As shown in Fig. 5J, both β-III tubulin and MBP were markedly up-regulated after Chase ABC treatment, consistent with the immunostaining results. However, the expression of GFAP in the treated group was not decreased as shown in the GFAP immunostaining. As the astrocytes differentiated from NSCs in Chase ABC treated group owning longer and more highly branched processes, we speculated that they might express more GFAP than those in the control. Taken together, these results suggest that CSPGs, whether expressed on the surface of NPCs or secreted into extracelluar matrix, may play an important role in controlling the differentiation of NPCs by some unknown direct or indirect mechanisms. Abolishing the function of CSPGs therefore may change the differentiation pattern of NPCs.

**Figure 6:**
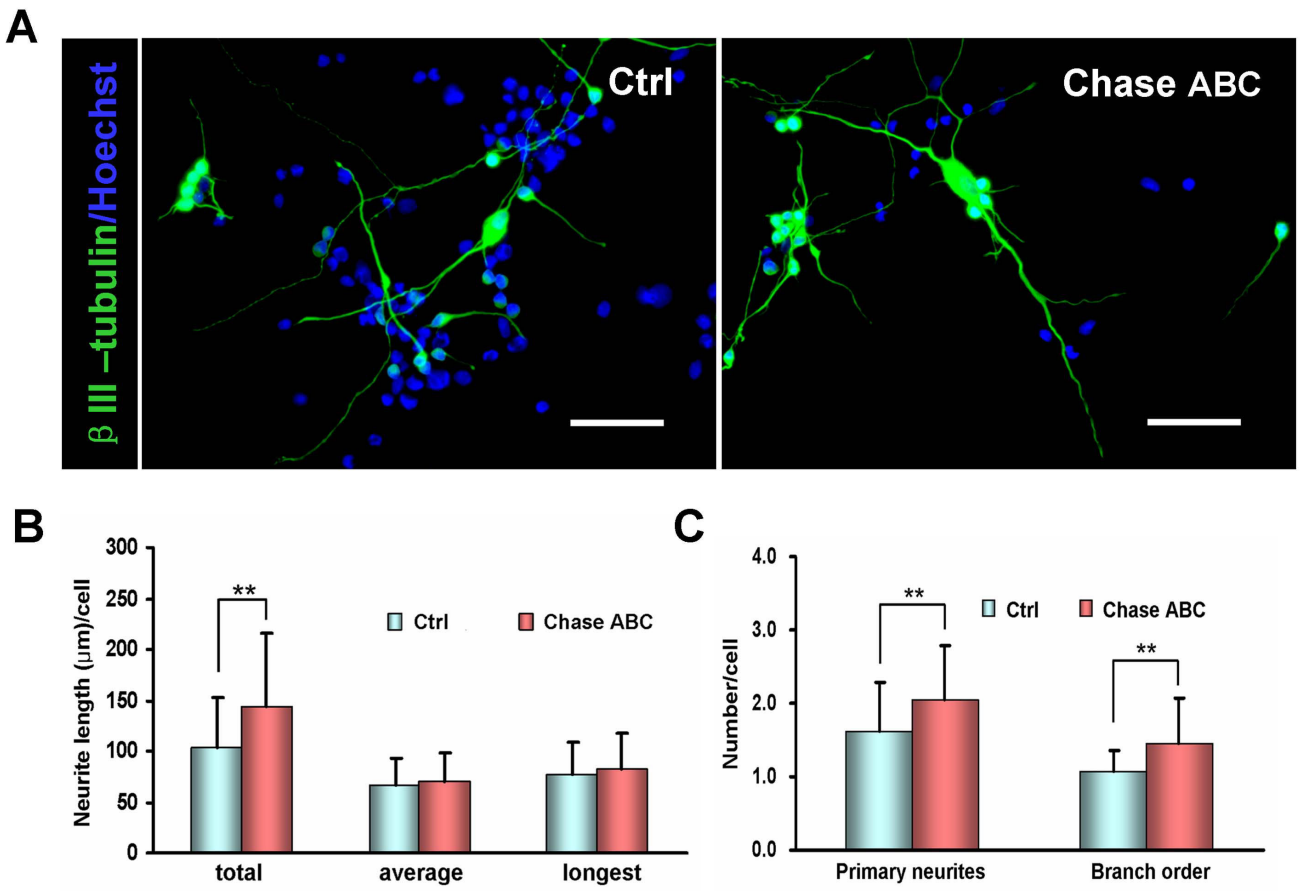
**Effects of Chase ABC treatment on neurite outgrowth of neurons derived from NPCs**. (A) NPCs were differentiated in 1% FCS for 5 days in the presence or absence of Chase ABC to allow differentiation into neurons with concomitant extension of neurites recognized by β-III tubulin immunostaining. Nuclei were counterstained with Hoechst 33342. Scale bar: 50 μm. (B) Total, average and the longest neurite lengths of each NPC-derived neuron were determined by Neurolucida software. The total neurite length per cell in Chase ABC treated group was longer than that in control, but no differences were found in average neurite length and the longest neurite length between the two groups. (C) The NPC-derived neurons in Chase ABC treated group showed more elaborate networks of neurites, including the number of primary neurite and the branch order, than the control. Data in B and C were measured with Neurolucida system and expressed as the mean ± SEM of four separate experiments with a total of 50 neurons examined in each group. **P < 0.01, treated vs control.

As Chase ABC treatment could significantly increase neuronal differentiation from NPCs, we further compared the neurites outgrowth in neurons differentiated from NPCs using β-III tubulin immunostaining at early stages of neuronal differentiation (green, Fig. 6A). Neurite outgrowth was analyzed by measuring the total, average and longest neurite length per cell, the number of primary neurites extending from the cell body, and the order number of branch points per cell. The results showed that neurons in Chase ABC treated group had greater total neurite length (143.9 ± 72.5 μm vs 102.7 ± 50.7 μm, P < 0.01), more primary neurites (2.04 ± 0.75 vs 1.63 ± 0.67, P < 0.01) and higher order number of branch points (1.46 ± 0.61 vs 1.08 ± 0.28, P < 0.01) than those in the control (Fig. 6B, C). The average neurite length and the length of the longest neurite of each neuron in the treated group were slightly longer than that of the control but the difference was not statistically significant (70.1 ± 28.9 μm vs 67.2 ± 25.7 μm; 81.8 ± 35.7 μm vs 77.8 ± 31.1 μm, respectively, both P > 0.05; Fig. 6B). These results suggest that digesting the CSPGs on NPCs or in ECM not only increases the neuronal differentiation from NPCs but also enhance neurite outgrowth from these neurons.

### Integrins mediated the changes in the growth and differentiation of NPCs after exposure to Chase ABC

**Figure 7:**
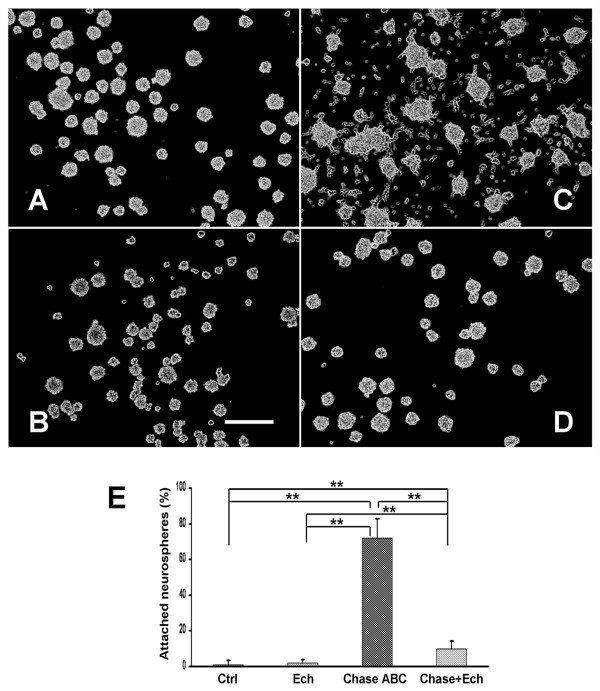
**Effects of Chase ABC on NPC morphology blocked by Echistatin (Ech)**. (A) A phase-contrast photomicrograph shows that floating neurospheres were formed from dissociated NPCs when cultured for 24 hours in the growth medium containing EGF and bFGF. (B) Application of Ech alone did not affect the morphology of neurospheres. (C) Exposure to Chase ABC induced neurospheres to attach to the bottom of the well and migration of cells outward. (D) Ech administration blocked the effects of Chase ABC in that NPCs maintained their inherit growth properties. Scale bar: 200 μm. (E) Quantitative data demonstrated that Chase ABC treatment resulted in a significant increase in the percentage of attached neurospheres, which could be blocked by the administration of Ech. Data were shown as mean ± SEM of four independent experiments with at least 350 neurospheres examined in each group. ** P < 0.01.

It was suggested that CSPGs in the CNS might function by directly or indirectly binding the integrins on the surface of neural cells [30], moreover, NPCs was reported to express several integrins on their surface [10]. To gain insight into the regulation mechanism of CSPGs on the biological properties of NPCs and determine whether integrin signal pathways are involved in the regulatory function of CSPGs on NPCs, we examined the influence of Chase ABC on NPCs properties in the presence or absence of Echistatin (Ech), the most potent known inhibitor of integrin. We first studied the morphological changes of the NPCs under the treatment of Chase ABC and Ech (Fig. 7). We found that Ech (0.2 μM) alone did not induce obvious changes in the morphology of neurospheres at 24 hours post-administration. The dissociated NPCs still grew and formed floating neurospheres, though a little small and the cell vitality was a little poor (Fig. 7B). As anticipated, Chase ABC (10 mU/mL) treatment significantly increased the percentage of attached neurospheres compared to the control (72.02% ± 10.89% vs 1.20% ± 2.16%, P < 0.01; Fig. 7C, E). But when both Chase ABC and Ech were administered, Ech could significantly reverse the effect of Chase ABC on the morphology of neurospheres, the percentage of attached neurospheres was decreased to 9.86% ± 4.53% (P < 0.01, Fig. 7D, E).

**Figure 8:**
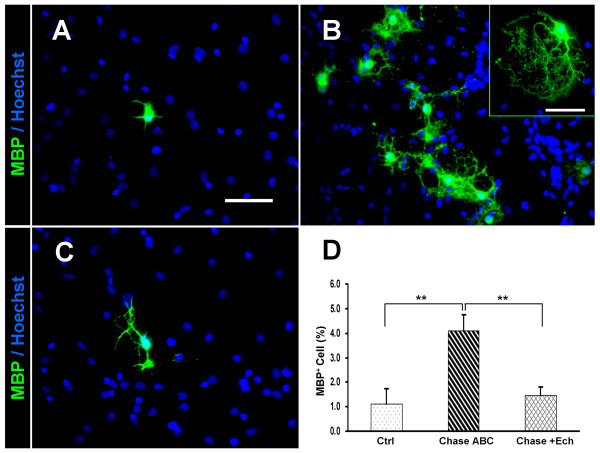
**Ech blocked the effect of Chase ABC on the differentiation of NPCs into oligodendrocytes**. (A-C) Representative immunofluorescence photomicrographs of oligodendrocytes (MBP^+^, green) differentiated from NPCs in the control (A), Chase ABC treated (B), and Chase ABC + Ech treated (C) groups. Hoechst 33342 (blue) was used to stain the nuclei. Scale bar: 50 μm. Boxed area in B showed mature morphology of an oligodendrocyte. (D) Quantitative data demonstrate that the Chase ABC treatment on NPCs resulted in a significant increase in the number of MBP^+ ^oligodendrocytes. Such an effect of Chase ABC could be markedly blocked by the administration of Ech. Data were shown as mean ± SEM of four independent experiments. ** P < 0.01.

A previous report revealed that the signal from integrins could regulate the morphological differentiation of oligodendrocytes [31]. As our result showed that Chase ABC treatment induced NPCs to differentiate into more MBP^+ ^mature oligodendrocytes, we asked whether the integrin inhibitor Ech could arrest the effect of Chase ABC on the differentiation of NPCs into mature oligodendrocytes. As anticipated, Chase ABC (10 mU/mL) treatment significantly increased the percentage of MBP^+ ^oligodendrocytes (4.09% ± 0.65% vs 1.10% ± 0.62%, P < 0.01; Fig. 8D) with more mature morphology (boxed area of Fig. 8B) compared to the control. While in Chase ABC and Ech (0.2 μM) treated groups (Fig. 8C), the percentage of MBP^+ ^oligodendrocytes was decreased to 1.45% ± 0.37% (P < 0.01) compared with Chase ABC treated group and returned to the control level (P > 0.05; Fig. 8D).

Thus, our results provide evidence that Ech can reverse the effects of Chase ABC on the morphology of NPCs and maturation of their progeny oligodendrocytes. As Ech is the most potent inhibitor of integrins, it suggests that CSPGs might be a native inhibitor of integrins. It also suggests that the integrin signal pathway may be involved in the regulation of CSPGs on the growth and differentiation of NPCs.

## Discussion

### The role of CSPGs on the biological properties of NPCs

The extracellular matrix (ECM) in the CNS plays a crucial role in neural development and neuropathology for its involvement in multiple developmentally- related processes including cell migration, neurite extension, synaptogenesis and synaptic plasticity. It has been well known that CSPGs are major components of the ECM in the CNS and major barriers to neural regeneration after traumatic injury in the adult CNS. However, like the other regeneration-inhibitory molecules, CSPGs are normal constitutive elements of the CNS and play physiological roles in the intact nervous tissue. Several lines of evidence indicate that CSPGs participate in different ontogenetic processes including neurogenesis and gliogenesis, cell migration, axon growth, and pathfinding [18,19]. In addition, the formation of CSPGs pericellular nets is associated with stabilization of synaptic contact and closure of critical periods [20].

Our present study demonstrated that CSPGs were also constitutively expressed on NPCs, which was consistent with a previous study identifying two isoforms of CSPGs phosphacan and RPTPβ on the surface of NPCs [32]. Another study also reported the presence of CSPGs in the NPC niches during late development and in the adult CNS [33]. Despite their morphological localization, little has been known about the function of these cell surface-associated molecules. In the present study, we used Chase ABC to degrade the CSPGs and determined the role of CSPGs on biological properties of NPCs. Our results demonstrate that digestion of CSPGs enzymatically resulted in substantial changes in morphology, migration and differentiation of NPCs. A striking morphological change was the significant reduction of their capacity to form neurospheres after Chase ABC treatment, consistent with the reports from Von HA and Sirko S [32,34]. But on the proliferation and differentiation of NPCs after Chase ABC treatment, our results were not completely identical with theirs [34], which might be due to different cell origin and detection methods. Kabos et al. reported that the expression of CSPGs on the NPCs was down-regulated with differentiation [35]. Though we did not detect the expression of CSPGs on NPCs and their lineage cells by Western blotting, and could not compare the expression of CSPGs among them, in this study, with double immunostaining, we found that CSPGs were also expressed on differentiated NPCs, which were consistent with our previous report [36]. Of course, the expression patterns of different kinds of CSPGs during NPCs differentiation remain to be explored.

As for the effect of Chase ABC treatment on the proliferation of NPCs, an important statement should be mentioned. Cell growth can occur via two different mechanisms: speeding up the rate of DNA synthesis in cells that have entered the S-phase; or raising the number of cells that have entered S-phase. The two assays ([^3^H] thymidine incorporation and cell cycle analysis) that we used could detect the two situations respectively and fully reflect the effects of Chase ABC on the proliferation of NPCs. Our results indicated that Chase ABC treatment did not change the cell cycle except the cells in G2-phase. However, as it affected the three dimensional structures of neurospheres, the NPCs in Chase ABC treated groups, due to moving out of the spheres, could uptake thymidine more easily and therefore enhance the growth of NPCs as shown in the results of [^3^H] thymidine incorporation. But we found that the treatment of 50 mU/ml Chase ABC did not significantly affect thymidine incorporation. In order to confirm the results from [^3^H] thymidine incorporation test, we also performed MTT test, a conventional method to evaluate the proliferation of cells cultured in vitro. The results of MTT assay showed that administration of Chase ABC at a dose of 0.5 and 5 mU/mL resulted in a significant increase in the proliferation of NPCs (P < 0.05), but at a dose of 0.05 and 50 mU/mL, no significant differences were observed (P > 0.05) (data not shown in detail). It indicated that the results from MTT were consistent with the outcome of [^3^H] thymidine incorporation test except the result of 0.05 mU/mL Chase ABC treatment group. As the samples treated with 0.05 or 50 mU/mL Chase ABC had relatively large standard deviations, we supposed that at lower and higher doses, it might be the actual experiments too variable to allow significant differences to be seen (Fig. 3A).

The other changes such as the migration and differentiation of NPCs after Chase ABC treatment might be a result of disaggregation of neurospheres. Although the exact relationship between neurosphere-forming cells in vitro and neural progenitor cells in vivo remains to be established, the neurosphere assay remains to be the best cell culture model for evaluating NPC biological properties. With that regard, the current results concerning the role of CSPGs on neurosphere forming in vitro should shed light on the behavior of neural progenitor cells in vivo. The previous studies revealed that the function of CSPGs was depended on their glycosaminoglycan (GAG) sulphated sugar chains. CSPGs can associate directly with cell surface adhesion molecules to enhance or block their function, and the probable mechanism is that the protein core of the CSPGs attaches to a matrix or cell surface molecule, bringing the highly charged GAG chain into a position in which it can mask or alter the structure of the target molecule, thereby affecting the downstream signal events and evoking the according changes of specific behaviors [37]. We believe that CSPGs may, in this way, play an important role in maintaining the morphology of NPCs in vivo and thereby regulate the migration and differentiation of NPCs during neural development.

### Interactions between CSPGs and integrins in maintaining the biological character of NPCs

One family of cell adhesion molecules that has been investigated in the context of brain development is the integrins, the primary mediator of neural cell behavior on ECM components. They are a major group of cell-surface receptors for both ECM and cell-surface molecules. The intergrins are composed of two non-covalently associated transmembrane glycoproteins, α and β, both of which participate in the binding of matrix proteins, and have been implicated in activating intracellular biochemical signaling pathways. For example, adhesion to ECM or clustering of specific integrins receptors has been shown to induce protein tyrosine phosphorylation, stimulate inositol lipid metabolism, activate G proteins, and enhance ion exchange across the cell surface [38,39]. ECM binding and integrin engagement also result in transmission of mechanical stresses across the cell surface that produce changes in cytoskeletal organization and associated alterations in cell shape [40-42]. In particular, the β1 family of integrins controls various nervous system cell functions, including survival [13], migration [14,15], neurite outgrowth [16], and myelination [17]. They have also been localized in various regions of the developing brain, and are present on the surface of each of the major cell types in the developing brain [15,43,44]. In addition, integrin gene deletion and antibody perturbation studies have demonstrated a functional role for integrin-ECM interactions in proper brain formation [14,45,46]. But the mechanisms by which such interactions contribute to fundamental NPC behaviors remain largely unexplored.

Previous reports showed that neurospheres were enriched in β1 integrins [15] and the expression level of β1 integrins conditioned the number of secondary neurospheres [12]. It is reasonable to speculate that the CSPGs bound on the surface of NPCs, like that in the ECM, through β1 integrins, modulate the behavior of NPCs by cell-cell, cell-ECM interaction or crosstalk between membrane receptors. To verify if integrins are involved in the function of CSPGs, we used the Ech, an inhibitor of integrins. Our results showed that Ech significantly reversed some effects of Chase ABC on CSPGs i.e. morphological change in neurospheres as well as maturation of oligodendrocytes derived from NPCs, while blocking integrins on NPCs alone did not significantly affect the formation of new neurospheres. These results were consistent with other reports in β1 integrin-deficient NPCs [47].

Our study also showed that, after exposure to Chase ABC, NPCs differentiated into more neurons, formed more complex neurite network, and increased the migration of neurospheres. Though we did not further explore the possible role of integrins beyond these phenomena, other researchers have reported that migration of β1 integrin-deficient neurospheres was significantly decreased on laminin substrates, and their adhesion was also disturbed [47]. These results suggest that the integrin signaling in the NPCs may be involved in some biological changes observed in the present study after the Chase ABC treatment. We speculate that the integrin on the surface of NPCs, by means of their ligands such as NCAM, Tenascin or other binding molecules, indirectly interact with the CSPGs bound on the cell surface or in the ECM and thus construct a well-defined microenvironment by crosstalk between membrane receptors or cell-ECM interaction, which regulates the NPC behavior. We reason that CSPGs may mask or alter the structure of integrins thereby inhibiting corresponding downstream signaling events making NPCs at a stable status which may be beneficial to the development of NPCs. Once we broke this balance by degrading CSPGs with Chase ABC, the integrins may be activated to induce a series of changes in the growth, adhesion, migration and differentiation of NPCs. When Ech is added, it inhibits integrins instead of CSPGs so that the condition of NPCs is stabilized. These results suggest that the integrin signal pathway might be involved in the regulation of CSPGs on some biological characters of NPCs though a mechanism remains to be investigated.

### Possible role of Chase ABC in mediating NPC-based cell transplantation

It has been well known that CSPGs are one of the inhibitory molecules found in glial scars and inhibit neurite outgrowth and induce growth cone collapse after spinal cord injury (SCI) [26,27] and Chase ABC application could promote axonal regeneration by digesting CSPGs in vitro [48] and in vivo [22,28,49,50]. In addition, NPC transplantation is another promising strategy which promotes axonal regeneration or neurite outgrowth by secreting neurotrophic factors, remyelinating or reconnecting the disrupted neuronal circuit [51-54]. Recently, researchers attempted to combine Chase ABC application with NPC transplantation to treat the injured spinal cord, and found this combined treatment significantly induced the outgrowth of a greater number of growth-associated GAP-43-positive fibers at the lesion epicenter, compared with NPC transplantation alone [25]. They attributed the enhanced effect to that Chase ABC attenuated the inhibitory effects of CSPGs around the lesion cavity and facilitated NPCs migration into host spinal cord. Results from our study have added an additional explanation of these effects, i.e. Chase ABC may have a direct effect on the growth and differentiation of transplanted NPCs.

In our studies, we demonstrate that treatment of Chase ABC did not inhibit the proliferation of NPCs nor their inherent cell cycle in vitro, which may be a prerequisite for the combination treatment of Chase ABC and NPC transplantation. But Chase ABC treatment changed the morphology of neurospheres and enhanced the migration of NPCs in vitro. So it remains to be investigated whether these changes would occur in vivo and benefit the migration of transplanted NPCs in the injured CNS.

An interesting observation is that Chase ABC induced more neuronal differentiation and less astrocytic differentiation from NPCs. If this also happens in vivo, then more neurons may be generated from the NPCs after Chase ABC application which may compensate for the loss of neurons following the CNS injury and may benefit to reconnect the disrupted neuronal circuit. Meanwhile, more mature oligodendrocytes differentiated from NPCs after Chase ABC treatment may enwrap demyelinated or regenerated axons and therefore contribute functional recovery after CNS injuries. Some investigators believe that pre-differentiation of NPCs into neurons or oligodendrocytes in vitro may be necessary to control the terminal lineage differentiation of the transplanted cells [55]. Our results suggest that combined application of Chase ABC and NPCs may partly avoid this process in obtaining favorable cell populations for repair. It should be noted that although the percentage of GFAP^+ ^astrocytes significantly decreased after exposure to Chase ABC, many of them showed aberrant morphology similar to those seen after brain disorders [56]. It is thus not clear whether these cells maintain their normal function or behave abnormally such as those seen after CNS injuries.

## Conclusion

Taken together, the present study investigating the influence and mechanisms of CSPGs on the differentiation and migration of NPCs should help us to understand the basic biology of NPCs during CNS development and provide new insights into developing new strategies for the treatment of the neurological disorders in the CNS.

## Authors' contributions

WLG designed and carried out the whole experiments, performed the statistical analysis and drafted the manuscript. SLF participated in the Immunocytochemistry. YXW and YL participated in the cell experiments. HZL participated in FACS analysis. PHL and XMX conceived the study, participated in its design and coordination and helped to draft the manuscript. All authors read and approved the final manuscript.
